# Effects of Football Training and Match-Play on Hamstring Muscle Strength and Passive Hip and Ankle Range of Motion during the Competitive Season

**DOI:** 10.3390/ijerph19052897

**Published:** 2022-03-02

**Authors:** Víctor Moreno-Pérez, Gil Rodas, Marcelo Peñaranda-Moraga, Álvaro López-Samanes, Daniel Romero-Rodríguez, Per Aagaard, Juan Del Coso

**Affiliations:** 1 Sports Research Centre, Miguel Hernandez University of Elche, 03202 Elche, Spain; vmoreno@umh.es; 2 Medical Department, Futbol Club Barcelona, 08028 Barcelona, Spain; gil.rodas@fcbarcelona.cat; 3 Elche Football Club SAD, 03208 Elche, Spain; david.penaranda@goumh.umh.es; 4 Faculty of Health Sciences, Universidad Francisco de Vitoria, 28223 Pozuelo de Alarcón, Spain; alvaro.lopez@ufv.es; 5 Sport Performance Area, Futbol Club Barcelona, 08028 Barcelona, Spain; danirrphysco@gmail.com; 6 Department of Sports Science and Clinical Biomechanics, Research Unit for Muscle Physiology and Biomechanics, University of Southern Denmark, 5230 Odense, Denmark; paagaard@health.sdu.dk; 7 Centre for Sport Studies, Rey Juan Carlos University, 28943 Fuenlabrada, Spain

**Keywords:** soccer, muscle injury, fatigue, team sport, flexibility, elite athlete

## Abstract

Deficits in hamstring muscle strength and in hip range of motion (ROM) have been considered risk factors for hamstring muscle injuries. However, there is a lack of information on how chronic exposure to regular football training affects hamstring muscle strength and hip ROM. The aim of this study was to examine the longitudinal effect of football training and competition during a complete season on hamstring muscle strength and hip ROM in football players. A total of 26 semi-professional football players underwent measurements of isometric hamstring muscle strength and passive hip flexion/extension, and internal/external hip rotation (IR/ER) ROM during the football season (pre-season, mid-season, end-season). Compared to pre-season, hamstring muscle strength increased in the dominant (+11.1%, *p* = 0.002) and non-dominant (+10.5%, *p* = 0.014) limbs in the mid-season. Compared to mid-season, hamstring strength decreased in the dominant (−9.3%, *p* = 0.034) limb at end-season. Compared to the pre-season, hip extension ROM decreased in mid-season in the dominant (−31.7%, *p* = 0.007) and non-dominant (−44.1%, *p* = 0.004) limbs, and further decreased at end-season (−49.0%, *p* = 0.006 and −68.0%, *p* < 0.001) for the dominant and non-dominant limbs. Interlimb asymmetry for hip IR ROM increased by 57.8% (*p* < 0.002) from pre-season to mid-season. In summary, while hamstring muscle strength increased during the first half of the football season in football players, a progressive reduction in hip extension ROM was observed throughout the season. The reduced hip extension ROM suggests a reduced mobility of the hip flexors, e.g., iliopsoas, produced by the continuous practice of football. Consequently, hip-specific stretching and conditioning exercises programs should be implemented during the football season.

## 1. Introduction

Hamstring muscle injury is a very common type of injury in professional football (also known as soccer in some countries) [1]. A previous study showed that a professional football team of 25 players could expect between 5–7 hamstring muscle injuries per season [2]. Further, the incidence of hamstring muscle injuries in professional football increases over time, at a rate of 2.3% per year [2]. Hamstring muscle strain injuries cause an important loss of time from football training and competition, and result in significant impact on team performance, with long-term financial implications as a result of player unavailability [3]. Thus, preventing hamstring muscle injury in football might have a critical importance for overall football performance, and for the well-being of professional football players.

To implement preventive strategies during football practice, the identification of risk factors associated with hamstring muscle injury is paramount [4]. It is well recognized that the probability of suffering a muscle injury is determined by the interaction between a number of non-modifiable and modifiable extrinsic and intrinsic risk factors [5]. Amongst the modifiable risk factors, hamstring muscle strength asymmetry [6], hamstring muscle strength deficit [7], and low hamstring-to-quadriceps strength ratios [6] have been identified as factors that increase the likelihood of suffering a hamstring muscle injury in team sports. Furthermore, team players with restricted range of motion (ROM) at the hip [8] and at the ankle [9] appear to be at elevated risk of sustaining hamstring muscle injury. Altogether, this information suggests that screening of both muscle strength and flexibility in the lower limb, to detect players with deficits and asymmetries in these two risk factors, may be important to prevent hamstring muscle injury during football practice and match-play.

Despite the above mentioned risk factors, other studies have failed to associate hamstring muscle injury with strength and flexibility deficits [10,11]. The differences in the outcomes of these investigations might be attributable to variations in the methodology used for muscle strength and/or injury assessment [5], and the time of the season when hamstring muscle strength and hip and ankle ROM have been examined [12]. Interestingly, Noya-Salces and coworkers [13] reported that injury incidence progressively increased during the football season due to chronic fatigue developed by the continuous training and competition. The gradual reduction in physical performance in football players could be attributed to the physiological stress imposed during the competitive season [14], together with a reduction in the time allocated for muscle strength training [15]. However, there is a lack of studies describing the effect of an entire season on modifiable risk factors associated with hamstring muscle injury. Therefore, the aim of the present study was to examine the effect of regular football training and match-play during a complete season on hamstring muscle strength and hip and ankle ROM in well-trained football players. We hypothesized that hamstring muscle strength and hip ROM would be lower in the pre-season due to detraining effects caused by the transition period. Further, it was hypothesized that maximal muscle strength and hip ROM would increase from pre-season to mid-season due to the intensified and condensed period of match-play, and that these parameters would remain relatively constant during the latter half of the season.

## 2. Materials and Methods

### 2.1. Participants

A total of 26 semi-professional football players volunteered to participate in the investigation. Participants had an age (mean ± SD) of 20.1 ± 1.9 years, a height of 176.9 ± 0.1 cm, a body mass of 72.4 ± 6.1 kg, and a body fat percentage of 10.7 ± 0.8%. Overall, 18 (69.2%) players had right lower-limb dominance while 8 (30.8%) had left lower-limb dominance. The following criterions of exclusion were adopted: a) history of pain within the previous month prior to testing; b) not regular training during the month prior to testing; c) musculoskeletal lower limb injury in three months prior to testing. Goalkeepers were excluded from the analysis. Another three field players were also excluded from the study sample because they sustained serious injuries (> 28 days of recovery needed for full participation in training and competition) during the season. Initially, we carried out a sample size calculation by using previous data on isometric hamstring muscle strength, and the difference in this value between injured and uninjured football players [6,16]. Before the start of this investigation, all players were fully informed about the testing and a written informed consent was obtained. This investigation was performed in accordance with the latest version of the Declaration of Helsinki 2013 and was approved by the Ethics Review Committee of the University.

### 2.2. Training Information

Players had been training an average of 13.0 ± 2.2 h/week across the full season, including all football training activities conducted in their clubs. The team’s physical coach collected in each training session and match the rate of perceived exertion (1–10-point scale) and the exercise time on an individual basis to calculate the session RPE (sRPE) [17]. The sRPE method takes into consideration both the intensity and the duration of a training session [18]. The week-long cumulative internal workload was calculated for each player by summarizing all sRPE scores recorded for the week (including the competition, measured in arbitrary units; a.u.). As part of their training activities, participants usually performed five days of field training which included physical conditioning exercises, small-sided games, skill-based routines, and tactical exercises. In addition, players performed a strength-based training program with free-weights with two macrocycles: the first one, mainly aimed to produce strength gains, was carried out from August to December, and included two strength training sessions per week. The training sessions in this first macrocycle consisted of four exercises (half-squat, bench press, seated leg curl, and seated row machine) with a load individually set at ~80% of participant’s one maximum repetition (1RM), and participants performed two series of 4–5 repetitions per exercise. The second macrocycle, which mainly aimed to maintain achieved strength levels, was performed from January to June, and included single weekly strength training sessions. The sessions consisted of four exercises (half-squat, lat pulldown, dead lift, and push press) at ~60% 1RM performed in two series of 6–8 repetitions per exercise. In both of these macrocycles, the strength-based session was carried using a standardized warm-up protocol that included 10 min of aerobic exercise (treadmill running or stationary cycling followed by two sets of six repetitions of bodyweight exercises, such as forward lunges, lateral lunges, submaximal jumps, push-ups, and trunk rotations, with a 15 s rest period between sets). All repetitions were performed at maximal intentional velocity for the concentric phase of the movement (with ~1 s for the eccentric phase and for the pause between repetitions), irrespective of the macrocycle or the exercise, with at least 3 min of recovery between series and between exercises. Additionally, all the strength-based sessions were carried out before the players performed a second training session within the same day consisting of field football-specific exercises.

### 2.3. Experimental Approach

In this prospective and observational investigation, testing was performed by two experienced members of the teams’ medical staff. During the whole season (10 months), the football players were tested at three different occasions: first week of the pre-season (July), which started after five weeks of a transition period with cessation of training [19]; at mid-season (first week of January), when players had competed 17 official matches; and by the end of the football season (last week of May), after completing the competitive season (38 official competitive games). At each time point (pre, mid, and end-season), players were evaluated for basic anthropometry including an estimation of fat mass by using the measurement of six skinfolds (triceps, subscapular, umbilicus, suprailium, thigh, and lower leg) and the formulae provided by Carter [20]. Afterwards, maximal isometric hamstring muscle strength, passive hip flexion ROM, hip extension ROM, hip IR ROM, and hip ER ROM were measured following this order. After this, ankle dorsiflexion ROM was assessed. All measurements were performed both in the dominant and non-dominant limbs, and they were performed in the morning (between 9:00 a.m. and 13:00 p.m.). One week prior to data collection, all study participants performed two separate familiarization trials to minimize the potential influence of learning effects on the outcomes of the investigation. In the case of the mid-season and end-season measurements, the measurement day was conducted at least 72 h after the last official match in order reduce the influence of fatigue in the variables under investigation [21].

### 2.4. Measurements

#### 2.4.1. Hamstring Muscle Strength

Maximal isometric hamstring muscle strength was measured by means of a handheld dynamometer (Lafayette Instrument Company, Lafayette, IN, USA), which was calibrated prior to each measurement. Prior to testing, all football players performed a standardized warm-up that consisted of 10 min of treadmill running, followed by two sets of six repetitions of forward lunges and lateral lunges, and two isometric repetitions of hamstring muscle contractions at ~50% and ~75% of self-perceived peak strength, separated by 20 s of recovery, and in the same position as the one used for testing. Carefully adopting previous procedures [22], three maximal voluntary contraction (MVC) attempts were performed for each limb, with a 30-s rest period between repetitions. During the MVC procedure the hip joint angle was 0°, while the knee joint angle was 15°, as previously recommended [23], and the dynamometer load cell was fixed 5 cm proximally to the lateral malleolus using a rigid strap. Each contraction was maintained at a maximal effort for 5 s, and the highest MVC value (*N*) in each trial was identified. Strong verbal encouragement was provided during all tests. The MVC attempt with the highest force value was selected for subsequent analysis. The intraclass correlation coefficient (ICC) for this test ranged from 0.86 to 0.88 [22].

#### 2.4.2. Hip Range of Motion (ROM)

Maximal range of motion (ROM) during passive hip flexion, hip extension, hip IR, and hip ER was measured using an inclinometer (ISOMED, Kirkland, WA, USA) with a telescopic arm, as described by Moreno-Pérez et al. [24]. Specifically, the inclinometer was placed approximately over the external malleolus for hip flexion ROM measurement, on the greater trochanter of the femur for hip extension ROM measurement, and the mid-point of the distal end of the fibula for hip IR and ER ROM measurements. In all of these measurements, the distal arm of the inclinometer was aligned parallel to an imaginary bisector line of the limb under testing. An assistant was responsible for proper stabilization of the pelvis during the measurements. The end point of each stretch was considered when the player felt a strong but tolerable stretch, before the onset of pain. Each measurement was performed twice for both legs with a 45 s rest period between measurements and limbs. The highest ROM value for each measurement was used in the subsequent analysis. The ICC for these tests ranged from 0.86 to 0.97 [25,26].

#### 2.4.3. Ankle Dorsiflexion

Unilateral ankle dorsiflexion ROM was assessed as described by Calatayud et al. [27] using the Leg-Motion system test (LegMotion, Check your Motion, Albacete, Spain). For this measurement, football players were in a standing position with the tested foot on the measurement scale and with their hands on their hips. The contralateral foot was positioned out of the platform with toes at the edge of it. Each participant performed the test with the assigned foot on the middle of the longitudinal line of the measurement scale, and just behind the transversal line that indicates the “zero” position. On command, participants were instructed to flex forward the knee to contact a metal stick of 70 cm of height, which was moved on the measurement scale until the participants could no longer maintain the heel on the ground. The distance obtained from the “zero” position to the metal stick was measured and recorded. A total of three repetitions were performed in each limb with 10 s of passive recovery between trials. The best score (largest ROM) among these measurements was selected for subsequent analysis. The ICC of the LegMotion System test was 0.96 to 0.98 [27].

### 2.5. Statistical Analysis

All statistical analysis was performed using the SPSS package (version 25, SPSS Inc., Chicago, IL, USA). Descriptive statistics including means and standard deviations were calculated for players’ descriptive characteristics and for the isometric hamstring muscle strength, passive hip flexion, extension, IR, ER, and ankle dorsiflexion ROM. Furthermore, in each player, the ROM (hip flexion, extension, IR, ER, and ankle dorsiflexion ROM) scores were categorized as either normal or restricted, according to reference values previously reported as clinically meaningful: for passive hip flexion ROM (<80°) [28], for hip extension (<0°) [29], for hip IR (<25°) [30], for hip ER (<25°) [31], and for ankle dorsiflexion (difference >2 cm between ankles) [32]. Chi-square tests (χ^2^) were used to compare the frequency of participants distributed in each category over time. Between-limb asymmetry was calculated as the percentage difference between limbs [33]. Normality of data distribution was verified using the Kolmogorov–Smirnov test. One-way repeated measures ANOVA was used to identify differences among the three moments of measurement. When a statistical significance was identified in the ANOVA, the Bonferroni post hoc test were applied to detect pairwise differences. Statistical significance was set at *p* < 0.05. In addition, to determine the magnitude of differences, Cohen’s effect size (*d*) and 95% confidence intervals (CI) were calculated and interpreted as: <0.2 trivial; 0.2–0.6 small; 0.6–1.2 moderate; or >1.2 large [34].

## 3. Results

**Figure 1:**
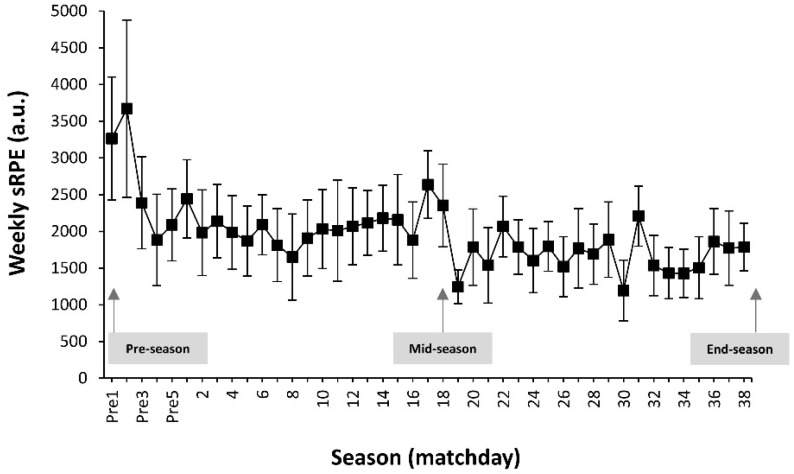
Week-long accumulated values of the session rate of perceived exertion (sRPE) across the season. Pre = indicates weeks without official competitions during the pre-season. The arrows indicate the moment of strength and range of motion measurement.

Football players competed, on average, of 17 ± 10 official matches (5–38 matches), with a total match playing time of 1465 ± 964 min (range = 68–543 min). Figure 1 depicts the evolution of the week-long cumulative internal workload across the season. Overall, there was a tendency for a progressive reduction on the values of sRPE.

Hamstring muscle strength values in the pre-season, mid-season, and end-season are presented in Table 1. Comparing pre-season to mid-season, hamstring muscle strength significantly increased in both the dominant limb (+ 11.1%, *d* = 0.617 [0.159, 1.037], *p* = 0.002) and in the non-dominant limb (+10.5%, *d* = 0.545 [0.098, 0.960], *p* = 0.014). In contrast, a decrease in hamstring muscle strength was observed from mid-season to end-season in the dominant limb (−9.3%, *d* = −0.442 [−0.008, −0.850], *p* = 0.034). No statistically significant changes were observed in dominant and non-dominant isometric hamstring muscle strength values from start pre-season to end-season (Table 1).

Table 2 shows values of the passive hip flexion, extension, IR, and ER, together with ankle dorsiflexion ROM throughout the season. A decrease in dominant and non-dominant hip extension ROM was found from pre-season to mid-season (dominant limb = −31.7%, *d* = −0.368 [−0.057, −0.722], *p* = 0.007; non-dominant limb = −44.1%, *d* = 0.519 [−0.075, −0.931], *p* = 0.004). A further decrease in the dominant and non-dominant hip extension ROM was found in the end-season measurement (Table 2). Furthermore, hip ER ROM showed a significant decrease from mid-season to end-season (−3.0%, *d* = −0.269 [−0.146, −0.670], *p* = 0.022). In contrast, higher values were observed from pre-season to mid-season in hip IR ROM in the non-dominant limb (+8.7%, *d* = 0.645 [0.183, 1.068], *p* = 0.028). No changes were observed in the remaining ROM measures, including passive ankle ROM at any time point of the season.

Table 3 depicts bilateral differences in the investigated variables. The only change during the season was an increase in the side-to-side difference for hip IR ROM from pre-season to mid-season (+57.8%, *d* = 0.795 [0.329, 1.262], *p* = 0.002).

## 4. Discussion

The aim of this study was to examine the longitudinal effect of football training and match-play during a complete football season on hamstring muscle strength and hip and ankle ROM in semi-professional football players. The rationale for this investigation was based on previous reports suggesting that weakness in hamstring muscles is an important risk factor associated with the development of hamstring muscle strain injury [7], with an additional contribution from reduced ROM at the hip [8] and ankle joints [9]. Confirming our hypothesis, hamstring muscle strength was lower in the pre-season than in the mid-season, but an unexpected decline in hamstring muscle strength was found from mid-season to end-season measurements, especially in the dominant limb. Additionally, our hypothesis about the maintenance of hip ROM variables during the latter half of the season could not be verified, as hip extension ROM decreased across the season for both the dominant and non-dominant limbs.

However, to the best of our knowledge, no previous investigation has evaluated the longitudinal modulation in hamstring muscle strength and hip/ankle flexibility across the competitive season of football athletes. The present data revealed that hamstring muscle strength increased at mid-season testing compared with pre-season levels in both the dominant and non-dominant limbs, while it returned to baseline levels by the end of the season. This outcome generally agrees with the finding of Wollin et al. [35], who reported that hip adductor strength and adductor/abductor strength ratio were lowest at pre-season, and they gradually increased during the weeks of intervention as players adapted to the demands of the training program. In the current investigation, players arrived from a five-week transition before the pre-season testing, while the transition period was characterized by complete cessation of all training activities, as previously suggested [19,36]. Interestingly, the workload of the weeks that conformed the pre-season period induced high ratings of perceived fatigue due to the detrained status of players, and due to the lack of lower intensity/lower volume sessions to prepare official matches or to recover from the match. This combination of factors ultimately indicated that the highest levels in weekly cumulative workload were reached in the preseason period (Figure 1). Overall, these data suggest that football players might be more prone to hamstring muscle injury during football practice in the pre-season phase due to initial detraining effects caused by the transition period, potentially resulting in reduced hamstring muscle strength, together with a higher training workload. Interestingly, the strength deficit may be overcome during the mid-season as a positive result of the football training and physical conditioning performed during this phase of the season.

On the other hand, a significant reduction in hamstring muscle strength of the dominant limb was observed at end-season compared with mid-season in the present group of football players. Providing a possible explanation for this reduction, the study participants may have experienced a physical deconditioning effect due to a reduced of overall training workload and particularly in the number of strength training sessions performed in the last part of the season (i.e., the last four weeks of the competitive season). Figure 1 indicates that a slight but progressive reduction in weekly training workload occurred during the season, assessed by a composite variable that included internal (RPE) and external (exercise time) loads. This reduction of the training workload was induced by the necessity of increasing the number of recovery sessions per week as the result of the chronic workload along with the accumulated number of official matches. Of note, as indicated in the methodology, the frequency of strength training sessions was also reduced from twice per week to once per week from February until the end of the season, together with a reduction in the load set per exercise. This information suggests that the football players examined in the present investigation might have experienced a physical deconditioning towards the end of the season as the result of reduced training load during training aimed to offset cumulative fatigue [14,15]. These data suggest that, in addition to the preseason, the end-season phase may increase the likelihood of suffering a hamstring muscle injury during football practice or match-play.

The present study observed a decrease in hip extension ROM from pre-season to mid-season that was further decreased in the end-season (Table 1). In addition, the current results showed a significant decrease of hip ER ROM from mid-season to end-season in the dominant limb. Interestingly, 6 out of 26 players (23%) developed a restriction in hip extension ROM in the mid-season, while this restriction was maintained at the end-season in these players. In addition, 7 out of 26 players (27%) presented restricted ankle dorsiflexion in the dominant limb at the end of season (Table 1). To our best knowledge, no previous studies have evaluated the longitudinal effects of football training and match-play within an entire season on hip ROM, although a single study exists for ankle dorsiflexion ROM [37]. A possible explanation for the present reductions in passive hip extension ROM in our group of football players across the competitive season might be due to adaptations in the muscle–tendon complex induced by the large number of high-force movements tasks performed during training and matches. Football is an intermittent sport where players often perform sport specific motor tasks at maximum intensity, such as rapid accelerations, decelerations, changes of direction, jumping, and landing tasks that all involve coupled eccentric–concentric muscle actions (so-called stretch-shortening cycles: SSC) [38]. It is well known that longitudinal exposure to high-intensity eccentric muscle actions increases the stiffness of muscles and tendons [39] and decreases joint ROM [40]. Thus, the massive involvement of SSC muscle actions during the football season could be an explanation for the reduction in hip extension ROM.

The observation of no alterations in ankle dorsiflexion ROM throughout the football season in the present study differ from report by Moreno-Pérez et al. [37], who found a progressive reduction in ankle dorsiflexion ROM when assessed during a competitive football season. This lack of agreement between studies may be due to differences in external load demands during the season. For instance, Moreno-Pérez et al. [37] included 40 professional players who performed 30.8 ± 9.9 matches per season, covering 2222 ± 844 min/season, whereas the present players demonstrated a much lower match load (17 matches, ~1440 min for players participating full time in all matches). Consequently, the magnitude of cumulative match loading might be a governing factor for the effect of competitive football on ankle dorsiflexion ROM.

Based on previous study reports [41,42], the existence of between-limb strength or/and ROM asymmetries are associated with a higher likelihood of developing lower-limb injuries in football players. The present study revealed a greater difference in hip IR ROM between dominant and non-dominant limbs from pre-season to mid-season. This result is possibly due to the asymmetrical participation of the lower limbs during football practice [38]. Specifically, football players typically repeat technical gestures of kicking, side cutting, and controlling the ball mainly using the dominant limb while the other limb is used to support the body weight during these actions [43]. Consequently, an increased focus should be made to maintain hip IR ROM in the dominant limb throughout the football season, especially in players who perform a large number of kicking actions during the season.

### Study Limitations

The current study has several limitations that should be acknowledged. Firstly, as the current study has been performed in a specific sample of football players with a defined match and training load, the present findings may not be readily extended to other populations of high-level team sport athletes. In addition, although all players underwent the same training program, there were large differences in the match load among players. Further, the present outcome variables were obtained relatively infrequent (three times) during the competitive season, and the measurement of quadriceps muscle strength was not included in this study. Future studies could benefit from assessing hamstring muscle strength and passive hip and ankle ROM using more high-frequent protocols, and by studying the fluctuations of quadriceps muscle strength and hamstring-to-quadriceps ratio throughout the season. Lastly, although the current study included the measurement of several potential risk factors for hamstring and groin injury, no prospective measurement of these type of injury were measured in the present study sample. Future studies should evaluate whether the fluctuations in hamstring strength and hip mobility presented here are associated with corresponding fluctuations in the incidence of hamstring and groin injury, particularly during the second half of the season.

## 5. Conclusions

In summary, hamstring muscle strength was observed to fluctuate substantially in semi-professional players across the football season. Overall, football training produced enhancements in hamstring muscle strength during the mid-season, while the pre-season and end-season periods both may represent periods with increased risk of hamstring muscle injury due to low levels of hamstring muscle strength. In addition, the current study found a gradual reduction in hip extension ROM during the competitive season, pointing towards a progressively increased hip flexor stiffness as a result of the accumulated volume of training and match-play. This latter observation might suggest an increased risk of groin injury towards the latter half of the season. Collectively, the present data confirm the necessity of prescribing a preventive injury program aimed at maintaining stable levels of hamstring strength and hip mobility throughout the season in order to prevent groin and hamstring injury [44,45], as players demonstrate fluctuations in these variables as the result of their exposure to football training and competition.

## Figures and Tables

**Table 1:** Isometric hamstring muscle strength assessed at pre-season, mid-season, and end-season.

Hamstring Muscle Strength	Pre-Season	Mid-Season	End-Season	Pre- vs. Mid-Season *p* Value Mean Difference *d*	Pre- vs. End-Season *p* Value Mean Difference *d*	Mid- vs. End-Season *p* Value Mean Difference *d*
Hamstring muscle strength in the dominant limb (*N*)	361.5 ± 14.3	406.6 ± 16.7	368.7 ± 15.4	0.002 45.09 [15.84, 74.35] 0.62 [1.04, 0.16] *moderate*	1.000 7.19 [−29.18, 43.56] 0.10 [−0.31, 0.500] *trivial*	0.034 −37.91 [−73.44, −2.37] −0.44 [−0.85, −0.01] *small*
Hamstring muscle strength in the non-dominant limb (*N*)	342.2 ± 14.4	382.3 ± 16.1	355.7 ± 15.3	0.014 40.09 [7.05, 73.14] 0.55 [0.10, 0.96] *small*	1.000 13.48 [−27.89, 54.84] 0.18 [−0.23, 0.58] *trivial*	0.108 −26.61 [−57.45, 4.22] −0.33 [−0.78, 0.10] *small*

Abbreviations: *d* = Cohen’s effect size: Values between brackets are 95% confidence limits for mean difference and *d*.

**Table 2:** Hip extension, external rotation, internal rotation, flexion, and ankle dorsiflexion ROM assessed at pre-season, mid-season, and end-season.

Range of Motion	Pre-Season	Mid-Season	End-Season	Pre- vs. Mid-Season *p* Value Mean Difference *d*	Pre- vs. End-Season *p* Value Mean Difference *d*	Mid- vs. End-Season *p* Value Mean Difference *d*
Hip Extension (°) (Dominant)	5.3 ± 0.9	3.6 ± 0.9	2.7 ± 1.0	0.007 −1.692 [−2.964, −0.421] −0.368 [−0.722, −0.057] *small*	0.006 −2.615 [−4.543, −0.668] −0.569 [−0.986, −0.118] *small*	0.611 −0.923 [−2.737, 0.891] −0.184 [−0.583, 0.225] *trivial*
Qualitative outcome				Normal (0) ^$^	Normal (6)	Normal (6)
Hip Extension (°) (Non-dominant)	5.5 ± 0.9	3.11 ± 0.8	1.7 ± 0.7	0.004 −2.462 [−4.212, −0.711] −0.519 [−0.931, −0.075] *small*	<0.001 −3.808 [−5.715, −1.901] −0.778 [−1.242, −0.314] *moderate*	0.127 −1.346 [−2.959, 0.267] −0.328 [−0.730, 0.093] *small*
Qualitative outcome *				Normal (4)	Normal (7)	Normal (3)
Hip ER (°) (Dominant)	57.4 ± 1.4	58.71 ± 1.2	56.9 ± 1.3	0.850 1.288 [−1.727, 4.304] 0.173 [−0.235, 0.572] *trivial*	1.000 −0.481 [−3.798, 2.836] −0.064 [−0.464, 0.338] *trivial*	0.022 −1.769 [−3.324, −0.215] −0.269 [−0.670, −0.146] *small*
Qualitative outcome *				Normal (0)	Normal (0)	Normal (0)
Hip ER (°) (Non-dominant)	55.9 ± 1.4	57.23 ± 1.2	58.0 ± 1.1	0.860 1.269 [−1.723, 4.261] 0.177 [−0.231, 0.576] *trivial*	0.594 2.096 [−1.972, 6.165] 0.293 [−0.125, 0.694] *small*	1.000 0.827 [−1.723, 3.377] 0.128 [−0.278, 0.526] *trivial*
Qualitative outcome *				Normal (0)	Normal (0)	Normal (0)
Hip IR (°) (Dominant)	46.4 ± 1.3	47.9 ± 1.1	48.13 ± 1.2	0.851 1.462 [−1.960, 4.883] 0.212 [−0.199, 0.611] *small*	1.000 1.692 [−2.942, 6.326] 0.246 [−0.168, 0.645] *small*	1.000 0.231 [−2.367, 2.828] 0.039 [−0.362, 0.439] *trivial*
Qualitative outcome *				Normal (0)	Normal (0)	Normal (0)
Hip IR (°) (Non-dominant)	47.4 ± 1.3	51.94 ± 1.4	51.09 ± 1.4	0.028 4.538 [0.410, 8.667] 0.645 [0.183, 1.068] *moderate*	0.108 3.692 [−0.579, 7.964] 0.525 [−0.080, 0.938] *moderate*	1.000 −0.846 [−3.066, −1.374] −0.111 [−0.509, 0.294] *trivial*
Qualitative outcome *				Normal (0)	Normal (0)	Normal (0)
Hip Flexion (°) (Dominant)	73.5 ± 1.2	72.0 ± 1.1	71.4 ± 1.0	0.296 −1.577 [−3.935, 0.781] −0.245 [−0.644, 0.169] *small*	0.123 −2.096 [4.592, 0.400] −0.326 [−0.750, 0.095] *small*	1.000 −0.519 [−2.105, 1.067] −0.089 [−0.488, 0.314] *trivial*
Qualitative outcome *				Normal (3)	Normal (5)	Normal (2)
Hip Flexion (°) (Non-dominant)	72.9 ± 1.2	71.7 ± 0.9	72.2 ± 0.9	0.748 −1.231 [−3.909, 1.447] −0.186 [−0.585, 0.223] *trivial*	1.000 −0.731 [−4.136, 2.674] −0.110 [−0.509, 0.294] *trivial*	1.000 0.500 [−1.418, 2.418] 0.102 [−0.300, 0.501] *trivial*
Qualitative outcome *				Normal (6)	Normal (6)	Normal (0) ^$^
Ankle dorsiflexion (cm) (Dominant)	11.0 ± 0.3	10.9 ± 0.3	10.4 ± 0.3	1.000 −0.173 [−0.898, 0.552] −0.090 [−0.489, 0.314] *trivial*	0.406 −0.663 [−1.767, 0.440] −0.346 [−0.748, 0.077] *small*	0.541 −0.490 [−1.403, 0.423] −0.309 [−0.711, 0.110] *small*
Qualitative outcome *				Normal (0) ^$^	Normal (2)	Normal (7) ^$^
Ankle dorsiflexion (cm) (Non-dominant)	10.8 ± 0.4	11.4 ± 0.3	11.0 ± 0.4	0.517 0.625 [−0.517, 1.767] 0.286 [−0.131, 0.686] *small*	1.000 0.231 [−1.140, 1.602] 0.105 [−0.299, 0.504] *trivial*	0.733 −0.394 [−1.242, 0.459] −0.212 [−0.611, 0.199] *small*
Qualitative outcome *				Normal (1)	Normal (1)	Normal (3)

Abbreviations: *d* = Cohen’s effect size: Values between brackets are 95% confidence limits for mean difference and *d*. * Qualitative score of the mean range of motion, in parentheses the number of players with a restricted range of motion score according to previously published cut-off scores (see Section 2.5); ^$^ = Depicts that the frequency of football players categorized as “normal” was different from the expected value at *p* < 0.05.

**Table 3:** Bilateral differences (dominant vs. non-dominant) for isometric hamstring muscle strength, passive hip flexion, extension, external rotation, internal rotation, and ankle dorsiflexion ROM assessed at pre-season, mid-season, and end-season.

Bilateral Difference (Dominant vs. Non-Dominant)	Pre-Season	Mid-Season	End-Season	Pre- vs. Mid-Season *p* Value Mean Difference *d*	Pre- vs. End-Season *p* Value Mean Difference *d*	Mid- vs. End-Season *p* Value Mean Difference *d*
Isometric Hamstring strength (%)	13.0 ± 8.1	12.2 ± 9.8	10.9 ± 8.8	1.000 −0.818 [−7.006, 5.371] −0.097 [−0.499, 0.304] *trivial*	0.846 −2.120 [−7.067, 2.826] −0.253 [−0.661, 0.154] *small*	1.000 −1.303 [−8.183, 5.578] −0.127 [−0.530, 0.274] *trivial*
Hip Extension ROM (%)	37.1 ± 34.0	30.6 ± 40.4	53.8 ± 39.4	1.000 −6.553 [−32.766, 19.660] −0.185 [−0.590, 0.218] *trivial*	0.424 16.707 [−11.516, 44.931] 0.473 [−0.048, 0.899] *small*	1.000 23.260 [−5.741, 52.262] 0.557 [−0.123, 0.991] *small*
Hip ER ROM (%)	6.5 ± 6.3	6.7 ± 7.1	8.89 ± 6.4	1.000 0.254 [−2.605, 3.112] 0.038 [−0362, 0.439] *small*	0.172 2.387 [−0.688, 5.462] 0.364 [−0.051, 0.779] *small*	0.238 2.133 [−0.859, 5.126] 0.289 [−0.120, 0.699] *small*
Hip IR ROM (%)	5.9 ± 5.2	10.3 ± 7.3	9.5 ± 7.0	0.002 4.341 [1.413, 7.269] 0.795 [0.329, 1.262] *moderate*	0.065 3.540 [−0.169, 7.249] 0.649 [−0.023, 1.094] *moderate*	1.000 −0.801 [−5.171, 3.569] −0.105 [−0.507, 0.296] *trivial*
Hip Flexion ROM (%)	4.0 ± 3.1	3.6 ± 3.9	4.0 ± 3.4	1.000 −0.346 [−1.850, 1.158] −0.105 [−0.507, 0.297] *trivial*	1.000 0.093 [−2.146, 2.331] 0.028 [−0.372, 0.429] *trivial*	1.000 0.439 [−1.857, 2.734] 0.108 [−0.293, 0.510] *trivial*
Ankle dorsiflexion ROM (%)	7.46 ± 7.2	10.0 ± 8.9	10.5 ± 6.8	0.794 2.586 [−3.241, 8.414] 0.344 [−0.069, 0.758] *small*	0.403 3.089 [−2.035, 8.214] 0.411 [−0.007, 0.831] *small*	1.000 0.503 [−5.197, 6.203] 0.054 [−0.346, 0.455] *trivial*

Abbreviations: *d* = Cohen’s effect size: Values between brackets are 95% confidence limits for mean difference and *d*.

## Data Availability

The data presented in this study are available on request from the corresponding author. The data are not publicly available due to restrictions of the club where the data were obtained.
